# Reliability of three foot models to examine paediatric gait

**DOI:** 10.1186/1757-1146-5-S1-O18

**Published:** 2012-04-10

**Authors:** Ryan Mahaffey, Stewart Morrison, Wendy Drechsler, Mary Cramp

**Affiliations:** 1 School of Health, Sport & Bioscience, University of East London, UK

## Background

A variety of multi-segmental foot models have been produced to examine patterns of foot segmental movement during gait cycle to identify biomechanical differences between normal and pathological foot function[1-3]. The reliability of foot models to accurately describe motion of the foot joints is dependent on the ability of the examiner to repeatedly apply markers to specific landmarks and the relevance of models’ segmental descriptions to underlying anatomy. The aim of this study was to test the reliability of segmental angles measured by three published foot models during paediatric gait.

## Materials and methods

Sixteen children, aged 6 to 12 years old, were recruited to the study. Marker sets for three foot models 3DFoot[1], Oxford Foot Model (OFM)[2], and Kinfoot[3] were applied to their right feet simultaneously which to the authors knowledge, is the first direct comparison of the three models during gait. Each foot model was assessed for repeatability of maximal joint angle and range of motion during the gait cycle between two testing occasions. Absolute angular differences and standard error of measurement (SEM) are reported.

## Results

Repeatability of all maximal segmental angles and range of motions were higher in 3DFoot compared to OFM and Kinfoot (Table 1).

**Table 1:** Inter-session repeatability of foot model’s 3D maximal segmental angles over the gait cycle.

Model	Segments	Maximal joint angle		Range of joint angle	
		° Difference	SEM °	° Difference	SEM °
OFM	Hindfoot to Shank	2.1 ± 15.1	10.9	1.2 ± 8.0	5.7
3DFoot	Hindfoot to Shank	1.0 ± 5.2	3.6	1.0 ± 4.6	3.3
Kinfoot	Hindfoot to Shank	1.0 ± 5.1	3.6	1.4 ± 6.3	4.3
3DFoot	Midfoot to Hindfoot	0.8 ± 3.5	2.2	0.3 ± 2.7	1.9
Kinfoot	Midfoot to Hindfoot	3.0 ± 11.1	6.7	3.7 ± 11.3	6.6
OFM	Metatarsals to Hindfoot	0.8 ± 8.5	5.3	1.3 ± 5.7	5.4
3DFoot	Metatarsals to Midfoot	0.7 ± 4.0	2.9	0.6 ± 3.6	2.5
Kinfoot	Metatarsals to Midfoot	2.8 ± 7.8	4.8	2.6 ± 6.6	3.7
OFM	Hallux to Metatarsals	2.3 ± 15.6	11.2	0.4 ± 13.7	9.1
3DFoot	Hallux to Metatarsals	1.5 ± 10.0	6.2	0.4 ± 12.6	8.8
Kinfoot	Hallux to Metatarsals	4.4 ± 21.8	15.1	2.1 ± 11.8	7.2

## Conclusion

Decreased measurement error observed in 3DFoot and Kinfoot models may be attributable to normalisation of kinematics data to subject standing position. In the OFM, non-normalisation of gait data resulted in variable segmental offsets, particularly in the frontal plane. Greater measurement error was observed for several foot segments in the Kinfoot model. This may be due to discrepancies in model segment definitions in relation to the underlying joint anatomy, especially around the midfoot to hindfoot segments. 3Dfoot model consistently showed the least measurement error in the segment motions examined and thus is appropriate for use to examine foot biomechanics in gait.
